# A fast and accurate identification model for *Rhinolophus* bats based on fine-grained information

**DOI:** 10.1038/s41598-023-42577-1

**Published:** 2023-09-29

**Authors:** Zhong Cao, Chuxian Li, Kunhui Wang, Kai He, Xiaoyun Wang, Wenhua Yu

**Affiliations:** 1 School of Electronics and Communication Engineering, Guangzhou University, Guangzhou, 510006 China; 2 School of Life Sciences, Guangzhou University, Guangzhou, 510006 China

**Keywords:** Computational biology and bioinformatics, Zoology

## Abstract

Bats are a crucial component within ecosystems, providing valuable ecosystem services such as pollination and pest control. In practical conservation efforts, the classification and identification of bats are essential in order to develop effective conservation management programs for bats and their habitats. Traditionally, the identification of bats has been a manual and time-consuming process. With the development of artificial intelligence technology, the accuracy and speed of identification work of such fine-grained images as bats identification can be greatly improved. Bats identification relies on the fine features of their beaks and faces, so mining the fine-grained information in images is crucial to improve the accuracy of bats identification. This paper presents a deep learning-based model designed for the rapid and precise identification of common horseshoe bats (Chiroptera: Rhinolophidae: *Rhinolophus*) from Southern China. The model was developed by utilizing a comprehensive dataset of 883 high-resolution images of seven distinct *Rhinolophus* species which were collected during surveys conducted between 2010 and 2022. An improved EfficientNet model with an attention mechanism module is architected to mine the fine-grained appearance of these *Rhinolophus*. The performance of the model beat other classical models, including SqueezeNet, AlexNet, VGG16_BN, ShuffleNetV2, GoogleNet, ResNet50 and EfficientNet_B0, according to the predicting precision, recall, accuracy, F1-score. Our model achieved the highest identification accuracy of 94.22% and an F1-score of 0.948 with low computational complexity. Heat maps obtained with Grad-CAM show that our model meets the identification criteria of the morphology of *Rhinolophus*. Our study highlights the potential of artificial intelligence technology for the identification of small mammals, and facilitating fast species identification in the future.

## Introduction

Bats are one of the most diverse groups of mammals, accounting for approximately one-fifth of all mammals^1^. They distribute widely throughout the globe except for the polar regions es and provide a variety of ecosystem services, such as pollination and pest control^2,3^. Bats have been shown to tolerate and survive many viruses that have high mortality rates in humans, such as SARS-CoV, SARS-CoV-2, MERS-CoV, Marburg, and henipaviruses^4^. Despite the ecological importance and research value of bats, their status is far from ideal. Habitat loss, urbanization, hunting, disturbance, and climate change have led to the listing of 51% of bat species as endangered or above^5,6^. One of the main obstacles is their extremely high species diversity and similarity in appearances. The identification of bats indeed is an extremely challenging task, and experts needed to make empirical judgments based on morphological character, body size, coloration of pelage, etc. These manual detection methods are costly and time-consuming, as well as professionally demanding. Although bioacoustic-based identification methods that take advantage of echolocation have been applied in species determination while their accuracy and precision are still criticized, and a promising application often relies on previous good surveys and precise species identification. Bioacoustic-based identification methods for bat species identification have been advantageous in the past; however, they require specialized equipment such as echolocation recorders and sophisticated detection processes, which may not always be practical in the field, particularly for real-time identification. Consequently, there is an urgent need for real-time bats identification methods based on the morphological features of bats. With the recent advancement of deep learning technology, it has become possible to achieve efficient and accurate animal identification using images. Computer vision-based identification methods are particularly useful for mining fine-grained information in images. The significant advantage of computer vision-based methods is their accuracy and efficiency compared to traditional manual methods, particularly in large-scale surveys and monitoring programs for bats.

In recent years, Deep learning has been increasingly utilized in various fields in recent year, one of its most successful application in image identification tasks^7^. In field of biological and medical research, deep learning has been employed in a range of tasks including cytopathologic analysis^8,9^, protein structure prediction^10^, gene relationship inference^11^, and physiological signal detection^12^. Biology researchers has been experimenting with the application of deep learning in animal species identification. Researchers attempted to use attributes such as their color^13^ and shape^14^ in images, achieving some success. Further studies, however, have shown that mining more complex and delicate features such as animal textures and contours, is crucial to improving identification and classification accuracy^15,16^. However, these methods did not fully exploit the fine-grained information hidden in the images. The convolutional neural network (CNN) model has shown significant promise in this regard, enabling model to distinguish objects even in a cluttered background. Freytag et al.^17^ used a Log-Euclidean CNN to predict chimpanzee attributes, such as identity, age, age group, and sex with considerable success. Deep learning models based on CNN have also been applied to identify animal species^18,19^, leading to a significant reduction in the time that taken by biological researchers and volunteers for such tasks. Willi et al.^20^ evaluated the performance of species identification using deep learning methods for camera images, and they found CNN achieved a considerable reduction in manual image identification time. Faster-CNN, a target detection framework with CNN, allows deep learning to be applied to a wide range of more diverse and complex scenarios during wildlife surveys^21^. The research on the application of CNN in animal species recognition indicates that they have high potential due to their strong feature extraction and discrimination capabilities. However, further research is still needed to develop image-based animal identification models that are fast, accurate, and better able to mine fine-grained information for real-time needs and high-similarity identification.

This study has a three-fold purpose: firstly, proposing a fast *Rhinolophus* bats identification model based on EfficientNet; secondly, improving the model accuracy by using the visual attention mechanism that can mine fine-grained information; thirdly, verifying the model meets the taxonomic key of *Rhinolophus*.

## Related works

### Convolutional neural network

Research in deep learning has made tremendous progress in recent years, especially in designing CNN architectures with higher accuracy for image recognition tasks. Typically, the approach to improve accuracy is to increase the depth of the CNN^22,23^, as it allows the model to learn and extract more complex image features. However, as the depth of the neural network increase, it becomes more challenging to optimize and prone to overfitting. Additionally, deeper networks have a larger number of parameters and require more memory and/or floating-point operations (FLOPs), making their development on mobile and embedded devices difficult. To address these challenges, lightweight CNN have increasingly applied in recent years^24,25^, which enable their development on the platforms with limited computing resources, such as mobile devices.

### EfficientNet model

Tan and Le^26^ proposed that designing mobile networks aiming toward higher accuracy and efficiency requires balancing the three dimensions of network width, depth, and resolution. They developed a multi-objective neural network that simultaneously explored the depth, width, and resolution, and resulted in an efficient network baseline called EfficientNet-B0. Using this baseline, they proposed an efficient compound method and generated a series of models from EfficientNet-B1 to EfficientNet-B7. As the versions of EfficientNet models applied from B0 to B7, the better the results obtained. Researchers can choose the versions that suit their needs for efficiency and accuracy for various tasks, such as lesion detection^27^, automated medical diagnosis^28^. EfficientNet has less parameters than some mainstream neural network models and achieves higher accuracy on image identification task, such as plant leaf disease classification^29^.

To enable real-time identification of *Rhinolophus* bats from Southern China, it is essential to use models that can easily run on embedded and mobile devices with limited computational resources. EfficientNet-B0 as a lightweight CNN, has parameters of only 5.3 M and FLOPs of 0.39G, making it suitable for deployment on mobile and embedded devices. We, thus, used the EfficientNet-B0 as our baseline model and was optimized for our identification tasks.

### Fine-grained visual identification

Fine-Grained Visual Identification (FGVI) is a challenging task because the differences between subcategories are usually small, so traditional manual identification methods are difficult to achieve the desired results. making traditional manual identification methods less effective. However, the advent of deep learning has led to significant improvements in this area, and FGVI was gaining more and more attention with the development of deep learning^30,31^. Some methods use visual attention mechanisms to capture the most salient attributes in images to improve the performance of neural networks for the FGVI task^32,33^. Such identification methods do not require rather additional steps and are highly scalable.

The identification of *Rhinolophus* bats from southern China usually relies on small variations in their nasal lobe shape, the size and shape of the auricle (a prominent part of the ear), and details of their fur and coloration. The use of fine-grained visual identification methods focuses more on these subtle features, resulting in automatic identification of *Rhinolophus* bats.

### Visual attention mechanism

The visual attention mechanism is an essential concept in modern deep learning, which was initially applied to the machine translation^34^ and later widely used in various vision tasks, such as image categorization^35^, target monitoring^36^ and medical image processing^37,38^. Visual attention can be explained in terms of the human biological system^39^, which is driven by cognitive (TOP-DOWN) factors such as goals, knowledge, and expectations that enable us to focus on the details of a visual scene and enhance the representation of key parts of the visual scene. Intuitively, the human visual system allows us to focus on salient areas in a cluttered scene and thus selectively focus on important information while ignoring secondary information, and it is this feature of the human visual system that is mimicked by the visual attention mechanism. In previous studies, several visual attention models had been proposed specifically for fine-grained visual categorization tasks and obtained good results, but the attention modules are computationally intensive, making them difficult to apply to mobile device^33,40,41^. Researchers thus prefer to design a small module that can be easily incorporated into a classical CNN architecture, which only requires a small amount of computational overhead to enable the neural network to learn.

In this study, we aim to develop a real-time identification of *Rhinolophus* bats that could be easily deployed on embedded or mobile devices. To achieve this, EfficientNet that used as a mobile network baseline for its powerful capabilities. Additionally, we incorporated an attention mechanism module, which could optimize EfficientNet and enables the network to learn more complex and richer features without adding computational overheads.

## Materials and methods

### Data acquisition and processing

**Figure 1:**
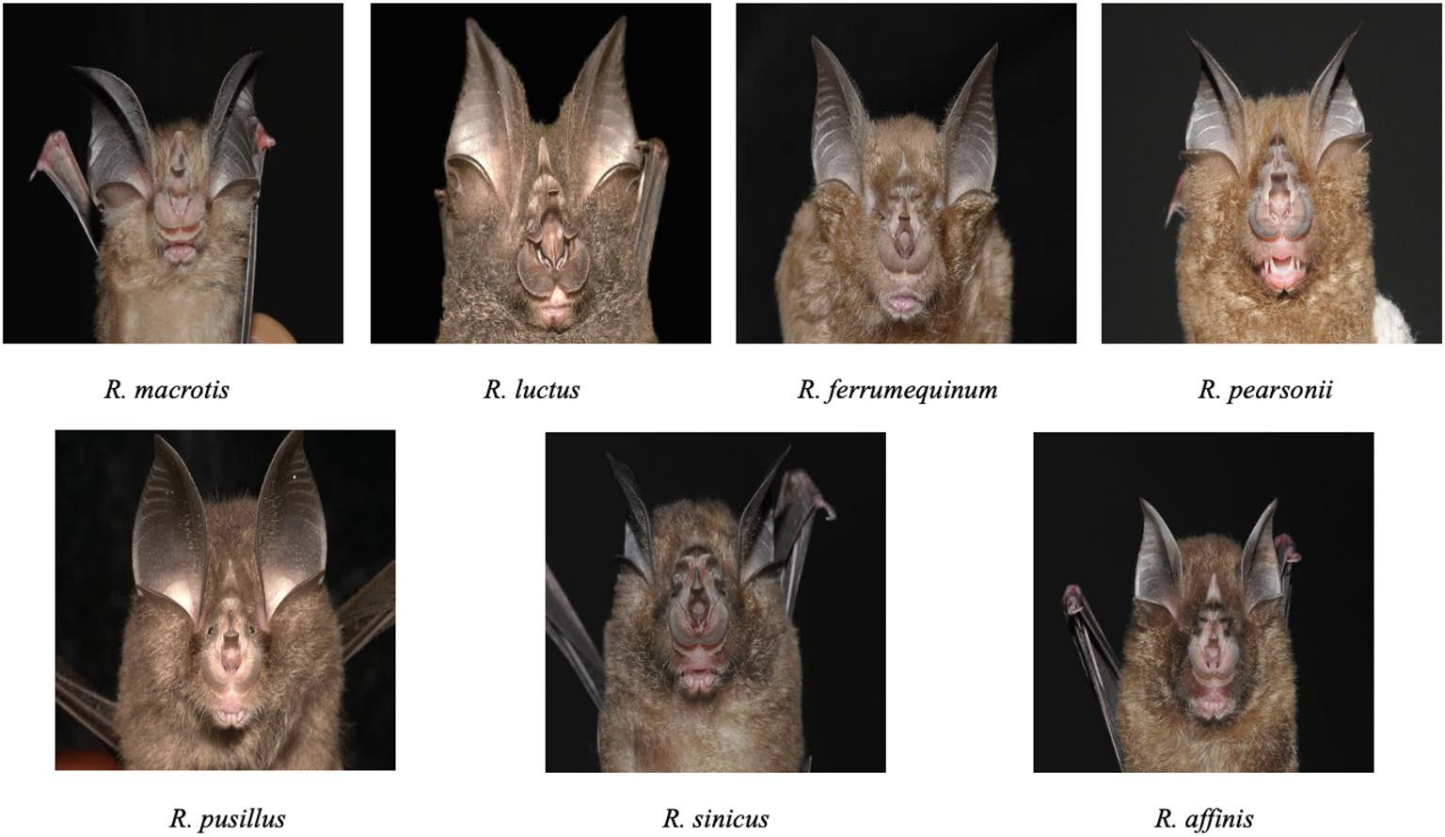
Examples of the dataset.

Bats are challenging species to observe or photograph as their unique habit including flying at night, and highly mobile, and do not have open-source datasets for species identification (e.g., ImageNet, Cifar). In this work, we collected images of *Rhinolophus macrotis*, *Rhinolophus luctus*, *Rhinolophus ferrumequinum*, *Rhinolophus pearsonii*, *Rhinolophus pusillus*, *Rhinolophus sinicus* and *Rhinolophus affinis* via a series of field surveys during 2010–2022 in South China. A total of 883 color images (RGB) of seven *Rhinolophus* bats were used as original dataset. The dataset was divided into a training set and a test set in the ratio of 2:1, with the test set containing 294 images. We illustrated some examples of the dataset in Fig. 1. They took the images at standard angles with minimal background variability to ensure the key features of *Rhinolophus*. These images are relatively pure and have less noise. It is also important to note that the number of images and the time of the shooting were not the same as when the images were taken due to the influence of bats. Despite these limitations, the dataset served as a starting point to apply deep learning and attention mechanisms to bat species identification.

### Data augmentation

The dataset collected for the study initially suffered from a data imbalance, with some species have relatively small sample sizes. This imbalance may lead to the lack of feature learning in the neural network. Therefore, we applied data augmentation method to address this issue and diversity of images^42^. In addition, data augmentation is a regularization tool to prevent the overfitting of neural networks, and a reasonable data expansion strategy can improve the generalization performance of neural network models^43^. We used the above-mentioned methods and randomly combined these methods for data augmentation to improve the overall performance of the dataset, including: (1) random cropping, random rotation, and flipping, a group of methods that can reduce the sensitivity of the neural network to the spatial location of the image; (2) brightness adjustment, hue change, and saturation adjustment, a group of methods that can reduce the sensitivity of the neural network to the image color; (3) random information discarding can reduce the neural network's dependence. These methods are effective in previous studies^23,44^ and are particularly critical in identification tasks with small data sets.

We also utilized a random information discarding method known as GridMask^45^. This method reduces the dependence of the neural network on individual local features of the horseshoe bat. After data augmentation, our training set was increased to 1318 images. The distribution of our dataset in experiments is presented in Table 1.

**Table 1:** Distribution of our dataset.

Class	Training	Test
*R. macrotis*	262	59
*R. luctus*	81	21
*R. ferrumequinum*	143	30
*R. pearsonii*	202	42
*R. pusillus*	268	62
*R. sinicus*	219	50
*R. affinis*	143	30

### Coordinate attention module

**Figure 2:**
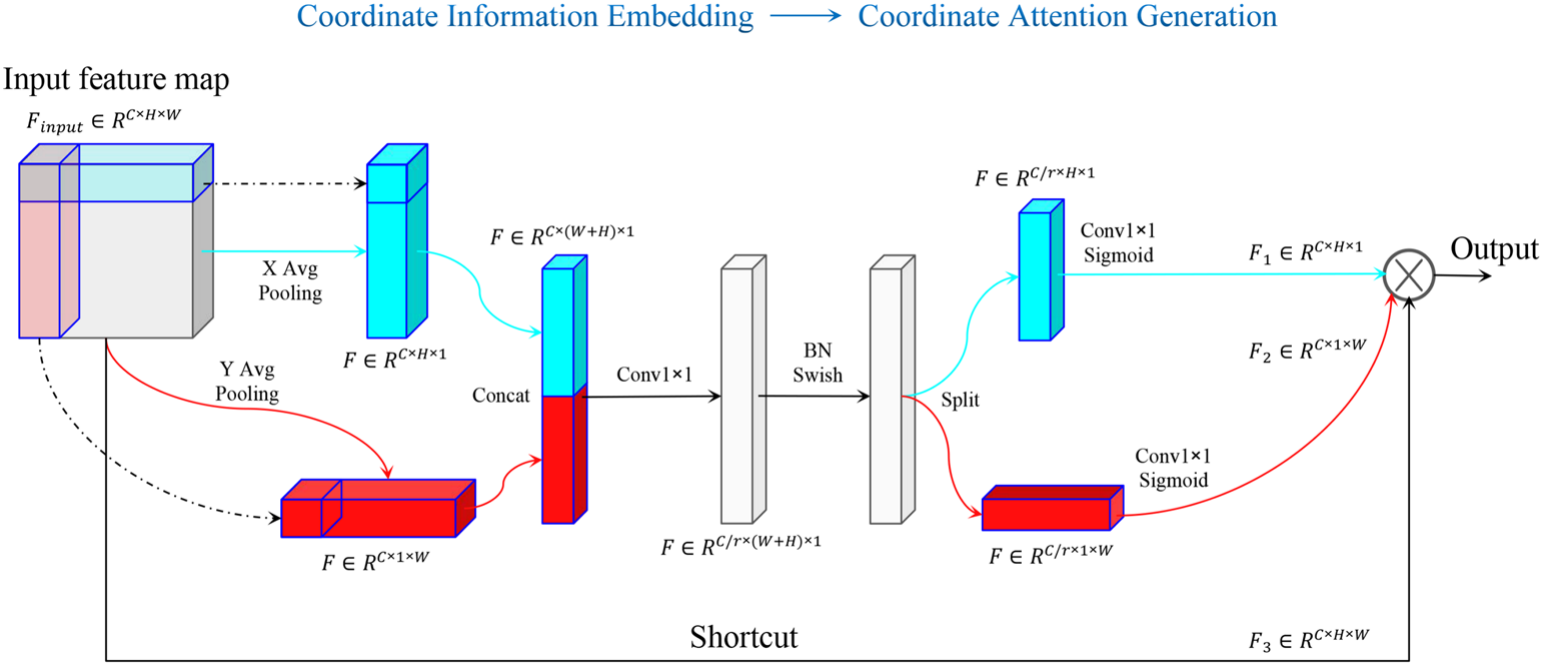
The structure of the CA module.

The main building block of EfficientNet-B0 is the mobile inverted bottleneck MBConv^46^ module with the attention block squeeze-and-excitation optimization^47^. However, EfficientNet-B0 ignores spatial attention, which is crucial for the localization of attention regions to allow the model to capture the structure of objects more efficiently. Hou et al.^48^ proposed a coordinate attention (CA) module, an efficient attention mechanism that decomposes channel attention into two parallel one-dimensional feature encoding processes to integrate spatial coordination information into the generated attention map. It captures cross-channel information, orientation-aware information, and position-sensitive information simultaneously, which facilitates the model to locate and identify key regions of the image, which improving our *Rhinolophus* bats identification accuracy. Meanwhile the CA module has almost no additional computational overhead, making it suitable for deployment on mobile devices without significant performance degradation. The CA module for the feature map has two stages, the Coordinate Information Embedding (CIE), and the Coordinate Attention Generation (CAG). The structure of the CA module is illustrated in Fig. 2.

The CIE phase of the CA module starts by calculating the global average of the input feature maps ${\varvec{X}}\in {\mathbb{R}}^{(C\times H\times W)}$ respectively along the height and width dimensions. This operation can be interpreted as a decomposition of the global average pooling in both the height and width directions, which allows the attention module to capture long-range dependencies along one spatial direction and retain positional information along another spatial direction. In the CAG phase, it concatenates the feature maps generated in the previous phase and feeds them to a shared $1\times 1$ convolutional operator function, which is equivalent to a learnable fully connected layer. Then, the obtained results are passed through a nonlinear activation function, which includes the operations of the Batch Normalization and Swish functions. In the next phase, the module separates the concatenated feature maps and transforms each of the two feature maps separately into a tensor with the same number of channels as the input feature maps using two separates $1\times 1$ convolutional transform functions. Finally, the module multiplies the two obtained feature maps with the input feature maps to generate the feature maps with CA information. Consequently, the CA module can locate objects more accurately, improving the overall performance of the model.

In this work, the addition of the CA module aims to improve the EfficientNet model's focus on the specific lobe-nose structures of the mouth, ears, and face of *Rhinolophus*, effectively utilizing positional information so that the model can better identify the unique features of each species and improve identification accuracy. Meanwhile, CA incurs minimal computational overhead, making it suitable for deployment on mobile devices without significant performance degradation.

### EfficientNet-CA model

**Figure 3:**
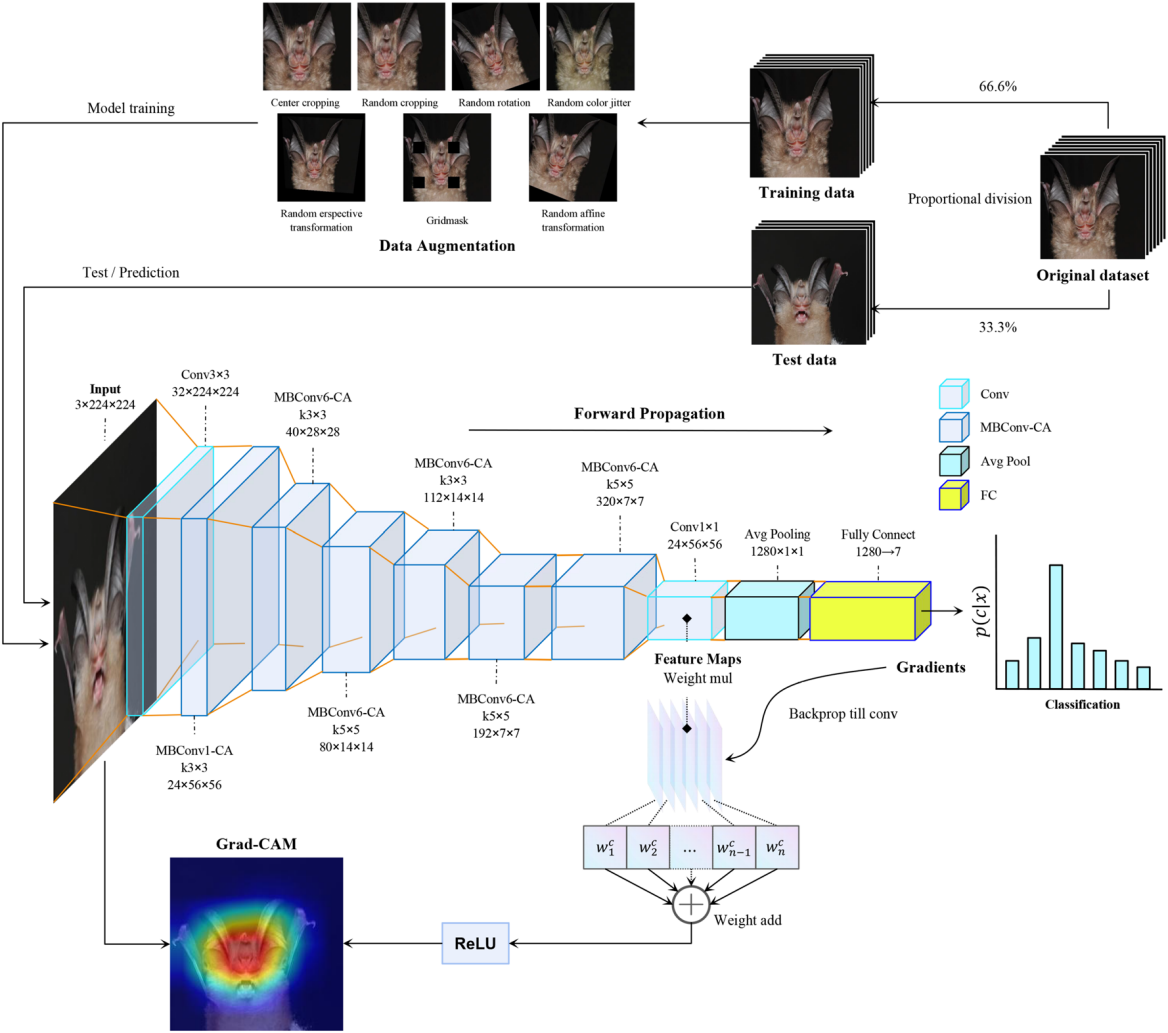
The schematic diagram of the proposed work.

In this work, the EfficientNet-B0 was used as our baseline model. To address the issues that squeeze-and-excitation optimization (SE) ignores spatial attention, we incorporated the CA module, which complements channel attention and embeds location information into channel attention. EfficientNet-B0 consists of building blocks mobile inverted bottleneck MBConv repeatedly stacked, and add the SE module. The MBConv module consists of a $1\times 1$ ascending convolution, depthwise convolution, SE block, $1\times 1$ descending convolution and dropout layer sequential connection, with residual connections before and after the module. In addition, some batch normalization layers and non-linear activation functions are included between their connections. We thus replaced all the SE blocks in MBConv with CA blocks to get the MBConv-CA model, they constitute the EfficientNet-CA. For the settings of EfficientNet-CA, we followed the default settings of EfficientNet-B0. The schematic diagram is shown in Fig. 3. Where, in MBConvN and $kn\times n$, $N$ denotes the ascending/descending ratio of $1\times 1$ convolution kernels, and $n$ denotes the Depthwise convolution kernel size. They follow the settings of EfficientNet-B0. The EfficientNet-CA model also contains a fully connected layer with seven species.

For the parameters of the CA module, we improved its computation of squeeze channels. The number of descent channels of improved CA module is shown by Eq. (1).

1 $${\mathrm{num}}_{\mathrm{squeeze}\_\mathrm{channel}}=\mathrm{max}(1,\mathrm{inc }// r),$$

where $inc$ denotes the number of input channels, $r$ denotes the reduction ratio, $r$ default is 24. In our task, the CA module of the early layers does not have to pay much attention to the resulting feature maps. On the one hand, it is because the simple features extracted in the early layer are not enough to provide help for bat recognition, and on the other hand, too much attention implies an increase in the number of parameters, and too many parameters will lead to a more difficult convergence of the CNN model. Therefore, our improvement reduces the CA module's focus on the feature map in the early layer and retains its level of focus on the feature map in the later layer. Our improved results will be verified in ablation experiments.

## Results

### Experiment setup and results

**Figure 4:**
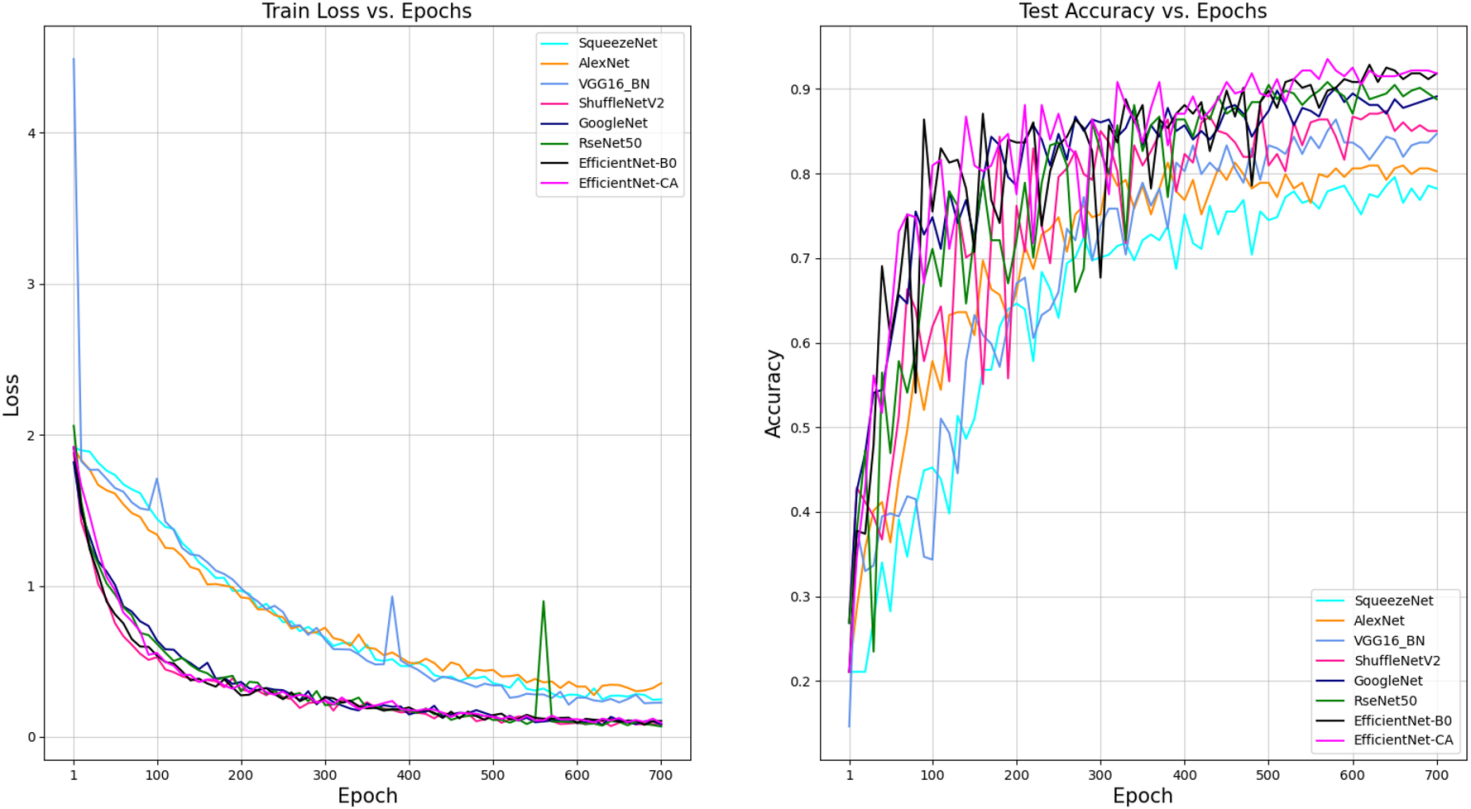
Training loss and testing accuracy of different models.

The PyTorch 1.3.1 framework was used to complete our experiments, including the construction and training of the model classifying our *Rhinolophus* species. The experimental program runs on NVIDIA TITAN V GPU and the batch size is set to 16. We trained the EfficientNet-CA model using the AdamW optimizer^49^ with a learning rate was ${5\times 10}^{-4}$, the weight of decay was ${5\times 10}^{-2}$, and a dropout probability of 0.2. For every neural network, we trained 700 epochs and saved the weights with the best performance. Figure 4 shows the loss of different CNN models on the training set and the accuracy on the test set. The experimental results surface that our proposed EfficientNet-CA has a fast convergence rate and fits the training set better. It is worth noting that EfficientNet-CA has the highest accuracy on the test set compared to other models.

### Evaluation metrics

Accuracy, precision, recall, and F1-score are all metrics used to evaluate a classification model’s performance. Accuracy represents the most intuitive indicator of a model’s classification performance and is defined as the ratio of the number of samples correctly predicted to the total number of samples predicted. Precision measures the ratio of true positive samples to total samples predicted positive. Recall measures the ratio of the number of true positive samples to the number of samples actually labeled as positive. F1-score is a weighted harmonic mean of precision and recall that takes into account both metrics. Equations (2), (3), (4) and (5) show the formulas for accuracy, precision, recall and F1-score respectively.

2 $$\mathrm{Accuracy}=\frac{\mathrm{TP}+\mathrm{TN}}{\mathrm{TP}+\mathrm{FP}+\mathrm{TN}+\mathrm{FN}},$$

3 $$\mathrm{Precision}=\frac{\mathrm{TP}}{\mathrm{TP}+\mathrm{FP}},$$

4 $$\mathrm{Recall}=\frac{\mathrm{TP}}{\mathrm{TP}+\mathrm{FN}},$$

5 $$\mathrm{F}1-\mathrm{score}=\frac{2*\mathrm{Precision}*\mathrm{Recall}}{\mathrm{Precision}+\mathrm{Recall}},$$

where $\mathrm{TP}$ indicates the number of samples predicted to be true positive, $\mathrm{FP}$ indicates the number of samples predicted to be false positive, $\mathrm{TN}$ indicates the number of samples predicted to be true negative, $\mathrm{FN}$ indicates the number of samples predicted to be false negative.

**Figure 5:**
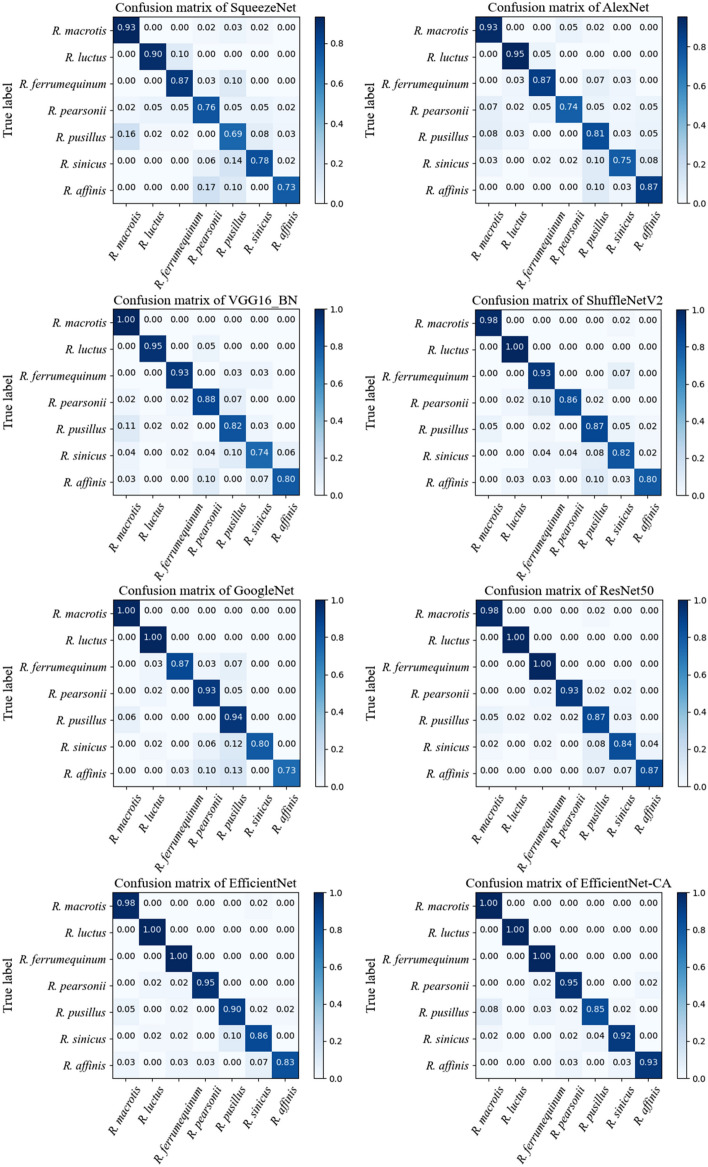
Confusion matrix of the predicted result of different models.

Evaluation metrics are essential to evaluate the classification performance of trained models. When the test set are imbalanced number of each species, the average accuracy may not reflect actual classification performance of the model. The confusion matrix has predicted species in each column and actual species in each row. The diagonal line represents the correctly predicted instances. The darker the color of the diagonal line indicates the accuracy of the classifier’s prediction. In our experiments, we compared the performance of our EfficientNet-CA model with other classical CNN models. We plotted the confusion matrix for the prediction results of different CNN models, as shown in Fig. 5. Our result shows that EfficientNet-CA achieves a prediction accuracy of over 90% for most our *Rhinolophus* species. Compared with other classical CNN models, EfficientNet-CA has only one prediction accuracy of less than 90% for only one species (*R. pusillus*), making the results more balanced and reliable.

Table 2 presents the evaluation values of our proposed EfficientNet-CA model and other CNN models on the prediction results in the *Rhinolophus* classification task. The results show that our proposed model outperforms other CNN models in our classification task. In addition, we also provide the evaluation results of EfficientNet-CA in predicting each *Rhinolophus* separately, as shown in Table 3. The experimental results show that EfficientNet-CA has a good classification effect on our candidate *Rhinolophus* species.

**Table 2:** Evaluation of the identification results of different CNN models.

Model	Precision	Recall	F1-score	Accuracy (%)
SqueezeNet^25^	0.813	0.810	0.810	80.27
AlexNet^50^	0.830	0.837	0.830	82.65
VGG16_BN^51^	0.883	0.876	0.877	87.07
ShuffleNetV2^52^	0.891	0.895	0.891	89.12
GoogleNet^23^	0.918	0.895	0.900	90.14
ResNet50^22^	0.924	0.927	0.925	91.84
EfficientNet-B0^26^	0.929	0.933	0.929	92.86
EfficientNet-CA	0.948	0.952	0.948	94.22

**Table 3:** Evaluation of the identification results of EfficientNet-CA.

Class	Precision	Recall	F1-score	Accuracy (%)
*R. macrotis*	0.91	1.00	0.95	100
*R. luctus*	1.00	1.00	1.00	100
*R. ferrumequinum*	0.91	1.00	0.95	100
*R. pearsonii*	0.93	0.95	0.94	95
*R. pusillus*	0.96	0.85	0.91	85
*R. sinicus*	0.96	0.92	0.94	92
*R. affinis*	0.97	0.93	0.95	93

### Performance comparison with other classical CNN models

To demonstrate the effectiveness and real-time performance of our approach, we compared the performance of EfficientNet-CA against other classical CNN models in the *Rhinolophus* bats identification task. As shown in Table 4, our customed EfficientNet-CA achieved the best accuracy, outperforming other lightweight CNN models. Although EfficientNet-B0 consumes more computational resources than these models, it did improve identification accuracy when comparing with other lightweight CNN such as SqueezeNet and ShuffleNetV2. Compared to EfficientNet-B0, the EfficientNet-CA with the addition of CA achieves higher accuracy with almost no increase in computational overhead. This makes it suitable for deployment on embedded and mobile devices with limited computational resources. ResNet50 has a deeper architecture and can capture more complex images features, leading to a good accuracy performance. However, it has 5 times more parameters and 10 times more FLOPs than EfficientNet-CA, and this computational overhead is unaffordable for embedded and mobile devices.

**Table 4:** Performance comparison of different CNN models in *Rhinolophus* bats identification.

Model	Params	FLOPs	Multi-Adds	Accuracy (%)
SqueezeNet	0.74 M	743.62 M	1.47G	80.27
AlexNet	57.03 M	711.48 M	1.42G	82.65
VGG16_BN	134.3 M	15.53G	31.0G	87.07
ShuffleNetV2	1.26 M	149.58 M	295.72 M	89.12
GoogleNet	5.61 M	1.51G	3.02G	90.14
ResNet50	23.52 M	4.12G	8.22G	91.84
EfficientNet-B0	4.02 M	398.03 M	789.3 M	92.86
EfficientNet-CA	4.34 M	408.98 M	810.77 M	94.22

Where Params denote the number of parameters of the model, FLOPs denotes the number of floating-point operations per second, Multi-Adds denotes the number of multiplications and additions, the unit M denotes ${10}^{3}$ and the unit G denotes ${10}^{6}$.

### Visualization of experimental results

**Figure 6:**
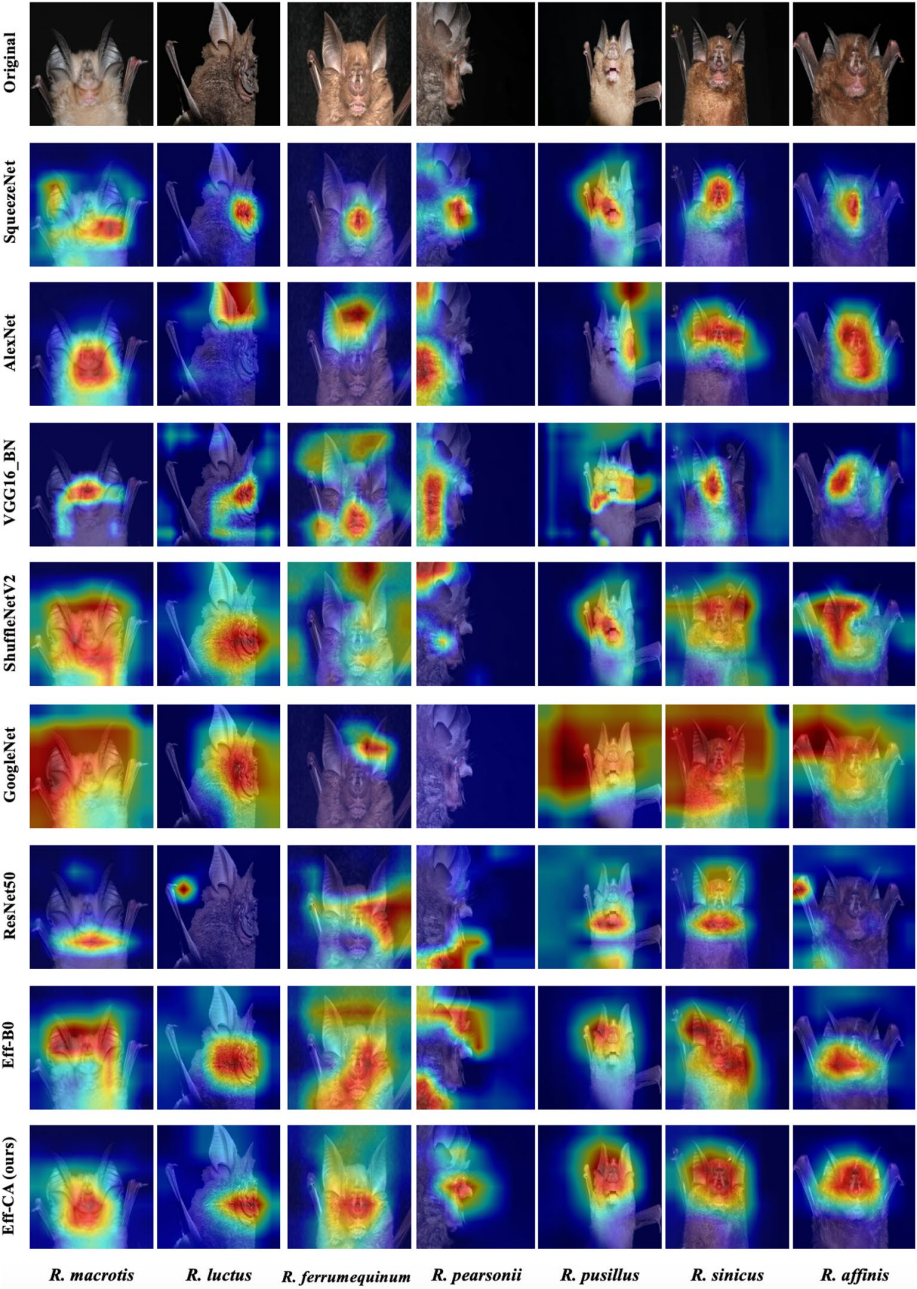
Visualization of feature maps produced in the last building block of different models.

To further validate the effectiveness of our proposed EfficientNet-CA approach, we employed Gradient-weighted Class Activation Mapping (Grad-CAM)to visualize the experimental results^53^. Grad-CAM is a visualization technique that highlights the importance of different regions in an image during identification by CNN model. By visualizing the activation maps of the last convolutional layer, Grad-CAM can help us understand where the CNN model is focusing while making its identification decision. In the *Rhinolophus* bats identification task the last convolutional layer of EfficientNet-CA and other CNN models were visualized via Grad-CAM (Fig. 6). The results illustrate that when comparing with other CNN models, EfficientNet-CA can focus more accurately on the nasal lobe and face, which are the key feature when classifying the *Rhinolophus* species by taxonomists.

### Ablation experiment

#### Advantages of data augmentation

To demonstrate the importance of data augmentation, we conducted a series of ablation experiments. We used the original dataset without data augmentation, which is still divided in 2:1 ratio, the distribution is shown in Table 5. Then, this dataset was used to train different neural networks, and the final experimental results are shown in Table 6. The experiments reflect that data augmentation strategy helps to improve the accuracy of different CNN models. Therefore, data augmentation is necessary.

**Table 5:** Distribution of original dataset.

Class	Training	Test
*R. macrotis*	120	59
*R. luctus*	36	21
*R. ferrumequinum*	61	30
*R. pearsonii*	86	42
*R. pusillus*	128	62
*R. sinicus*	100	50
*R. affinis*	59	30

**Table 6:** Comparisons of different CNN models before and after data augmentation.

Model	Accuracy (%)	
	Without data augmentation	After data augmentation
SqueezeNet	76.19%	80.27%
AlexNet	80.27%	82.65%
VGG16_BN	83.33%	87.07%
ShuffleNetV2	88.78%	89.12%
GoogleNet	90.14%	90.14%
ResNet50	91.16%	91.84%
EfficientNet-B0	89.80%	92.86%
EfficientNet-CA	92.18%	94.22%

#### Different reduction ratio $\mathrm{r}$ or descent channels

In order to verify the effectiveness of our improved CA modules, we constructed different EfficientNet-CA using CA modules with different reduction ratios for the experiments respectively, the corresponding results are shown in Table 7. Experimental results show that our improved CA module achieves the highest accuracy and further reduces the consumption of computational resources.

**Table 7:** Performance comparison of EfficientNet-CA constructed with CA modules for different descent channels.

CA module (reduction ratio $r$)	Params	FLOPs	Multi-Adds	Accuracy (%)
$r=16$	4.81 M	414.65 M	822.1 M	91.84
$r=24$	4.34 M	409.17 M	811.14G	93.88
$r=32$	4.11 M	406.47 M	805.75 M	92.52
$r=48$	3.88 M	403.85 M	800.52 M	92.18
Ours	4.34 M	408.98 M	810.77 M	94.22

#### Non-linear activation function selection

The choice of non-linear activation function usually has an important impact on the convergence speed and final performance of CNN model. Therefore, this part will explore the effects of some mainstream activation functions on the final accuracy of EfficientNet-CA, and the experimental results are shown in Table 8. In our experiments, we used the Rectified Linear Unit (ReLU)^54^, Leaky ReLU^55^, Mish^56^, and the Swish^57^ activation function by itself. Leaky ReLU avoids neuron death compared to ReLU. Mish is often used for target detection and has advantages such as low cost and good smoothness. Experimental results show that simply changing the activation function did not improve the performance of bat recognition.

**Table 8:** Comparisons of different activation function when taking EfficientNet-CA as the baseline.

Model + activation function	Accuracy (%)
EfficientNet-CA + ReLU	91.50
EfficientNet-CA + Leaky-ReLU	91.84
EfficientNet-CA + Mish	93.88
EfficientNet-CA + Swish	94.22

## Discussion

Deep learning is fast and effective for image recognition. However, FGVI tasks like bats identification are extremely challenging. Bats identification relies on the fine features of its beak and face. Therefore, mining fine-grained information in images is crucial to improve the accuracy of bat recognition. In this paper, we focus on a real-time identification method based on deep learning with *Rhinolophus* bats from Southern China as a practical example. We proposed an EfficientNet-based *Rhinolophus* bats identification method, which draws on the idea of coordinate attention to encoding location information, solving the squeeze-and-excitation optimization that ignores the problem of spatial attention. Our results demonstrated that our model achieves higher accuracy than other classical CNN models and better focuses on the fine-grained information of bats. The proposed method can be easily deployed on embedded or mobile devices with limited computing resources, enabling real-time identification of bat species for bat monitoring, survey, or science popularization tasks.

The main contributions of this paper are listed below: (1) We collated images of common horseshoe bats from Southern China and verified for the first time the feasibility of bat identification by means of images. (2) We propose an improved EfficientNet-CA network that achieves good performance in Rhinolophus identification. The method can be easily deployed on embedded or mobile devices with limited computing resources. (3) We provide an idea for real-time bat images identification. Firstly, bat images need to be taken for making the dataset. They took the images at standard angles with minimal background variability to ensure the key features of bats. Then, a CNN model is constructed to enhance the model's representation of bat-specific structures using positional information and fine-grained information in the image space. Finally, the trained CNN is deployed into an embedded or mobile devices.

We summarise the reasons for the good performance of EfficientNet-CA in the bat recognition task as follows: (1) EfficientNet-CA is effective thanks to its powerful baseline EfficientNet, which has strong feature extraction capabilities and is lightweight enough to be effective in the fine-grained visual identification (FGVI) tasks and mobile scenarios. (2) EfficientNet employs the Squeeze-and-Excitation optimization (SE) attention module, which improves the model's sensitivity to channel features but ignores spatial attention. Spatial attention helps the model to know “where to pay attention”. Compared to the SE module, the CA module complements the lack of spatial attention in the SE module, and therefore the recognition accuracy is improved. (3) Positional information is crucial to capturing target structures in visual tasks^58^. In bats identification, the specific lobe-nose structures of the mouth, ears, and face is the key to distinguish *Rhinolophus*. The CA module makes full use of the position information, which helps the CNN model to distinguish the specific structure of *Rhinolophus*. Therefore, EfficientNet-CA has good performance in the recognition task.

**Figure 7:**
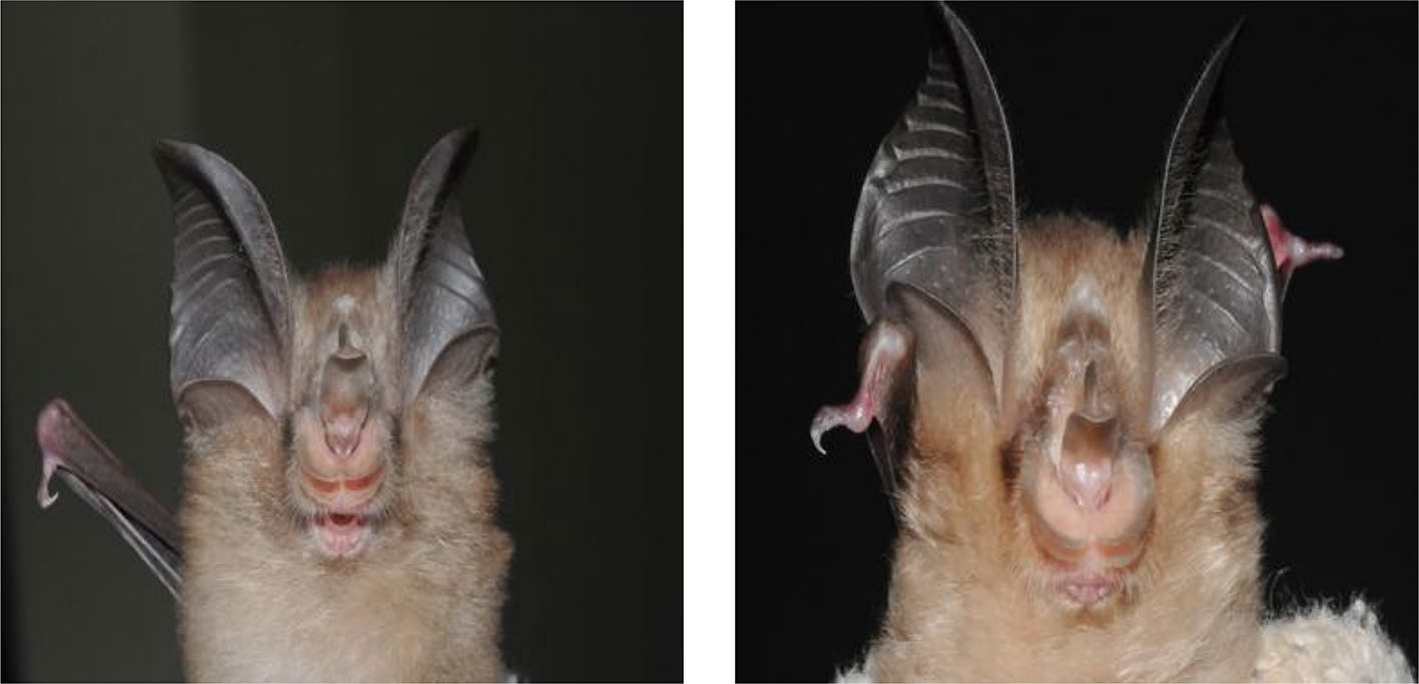
A comparative example of *R. macrotis* and *R. pusillus*.

The comparison of confusion matrices shows that most CNN models have low identification accuracy for *R. pusillus*. Most of the incorrect examples were incorrectly identified as *R. macrotis*. Actually, they do have great similarity in appearance and low distinguishability, as shown in Fig. 7. Non-image information such as body shape and size is the key to distinguishing them. Therefore, exploring multimodal information-based bats identification methods is the focus of our future work. Information is other than images but equally important should be used effectively.

Visualization of the experimental results shows that EfficientNet-CA generates better feature maps compared to other CNN models. From a mathematical point of view, given a feature map ${\varvec{X}}\in {\mathbb{R}}^{(C\times H\times W)}$, the SE module transforms the input feature maps into ${{\varvec{X}}}_{{\varvec{S}}{\varvec{E}}}\in {\mathbb{R}}^{(C\times 1\times 1)}$. This calculation loses the spatial features of the feature maps and retains only the channel features. Instead, the CA module decomposes channel attention into two parallel one-dimensional feature encoding processes to integrate spatial coordination information into the generated attention map ${{\varvec{X}}}_{{\varvec{H}}}\in {\mathbb{R}}^{(C\times H\times 1)}$ and ${{\varvec{X}}}_{{\varvec{W}}}\in {\mathbb{R}}^{(C\times 1\times W)}$. This calculation not only preserves the channel features, but also obtains information about the position of the input feature maps along the H and W directions.

For the real-time performance of the model, we analyze its possibility and potential for deployment on mobile and embedded devices only in terms of theoretical performance parameters. We did not actually deploy the model on these devices to study its effectiveness. Therefore, the deployment of mobile and embedded devices is also one of our future work priorities.

In the future, we will also investigate a deep learning model that can be used for larger-scale bat species identification. In addition, due to the current lack of open-source large-scale bat datasets in academia, dataset photography and collection remains a very important task.

## Data Availability

The data and code that support the findings of this study are available on https://github.com/czant1977/bat-identification.
